# Light Control of Ferromagnetism in ZnO Films on Pt Substrate at Room Temperature

**DOI:** 10.1038/srep45642

**Published:** 2017-04-10

**Authors:** Jihao Xie, Hongwei Qin, Yanming Hao, Bin Cheng, Weikang Liu, Liang Liu, Shaoqing Ren, Guangjun Zhou, Ziwu Ji, Jifan Hu

**Affiliations:** 1 School of Physics, State Key Laboratory for Crystal Materials, Shandong University, Jinan 250100, People’s Republic of China; 2 Department of Physics, College of Sciences, Tianjin University of Sciences and Technology, Tianjin 300457, People’s Republic of China

## Abstract

The control of ferromagnetism by light at room temperature is essential for the development of some optical-magnetic coupling devices, data storage and quantum computation techniques. In the present work, we demonstrate that the ferromagnetism of a semiconducting ZnO film on Pt substrate can be controlled by nonpolarized ultraviolet or violet light. The illumination of light with sufficiently high frequency photons could excite photogenerated electron-hole pairs in the semiconducting ZnO film. The amount of oxygen vacancies in the ZnO film and the appearance of built-in electric field due to the heterostructured ZnO/Pt may play important roles in the light-induced changes in the ferromagnetism of the ZnO film.

The control of magnetism using a non-magnetic field is one of the most attractive research subjects due to its potential applications in modern information technology. In addition to using electric fields and electric currents^1^,^2^,^3^,^4^,^5^,^6^,^7^, using light to control magnetism is one possible method that requires low energy consumption. The manipulation of magnetic properties by ultrashort laser pulses (including subpicosecond magnetization reversal^8^,^9^,^10^, optical generated coherent magnetic precession^11^,^12^ and laser-induced spin reorientation^13^,^14^) has attracted significant attention. Polarized light plays an essential role in the manipulation of the magnetic properties at the femtosecond time scale^15^. In fact, tuning the spin states by nonpolarized light has also been studied for a long time. A variation of the initial permeability under infrared radiation was observed in silicon-doped yttrium iron garnet at 20 K^16^, which is possibly due to the light-irradiated charge transfer from Fe^2+^ to Fe^3+^. The photo-induced ferromagnetism was observed in p-(In,Mn)As/GaSb and in several metal-organic assemblies, both at low temperatures^17^,^18^,^19^,^20^,^21^. For p-(In,Mn)As/GaSb, the carrier-mediated ferromagnetic interaction between the Mn ions is enhanced by the illumination of light through the generation of excess holes in the (In,Mn)As layer^17^. For metal-organic assemblies, the photo-induced ferromagnetism was attributed to either light-induced metal-to-metal charge transfer effect or light-induced excited spin-state trapping effect^18^,^19^,^20^,^21^. The optical control of anisotropic magnetoresistance in La_1/2_Sr_1/2_MnO_3-δ_ manganite was also investigated below 50 K^22^. It should be noted that these findings of nonpolarized light-controlled magnetism were observed at low temperatures^16^,^17^,^18^,^19^,^20^,^21^,^22^.

On the other hand, room temperature ferromagnetism has been found in many pure semiconducting or insulating oxides containing nonmagnetic elements in the forms of thin films or nanograins^23^,^24^,^25^,^26^,^27^,^28^,^29^. The observed room temperature ferromagnetism is called d^0^ ferromagnetism, which is believed to originate from some vacancies in the surface of the films or nanograins. Zinc oxide (ZnO), a direct wide-band-gap semiconductor, is suitable for use in a wide range of optical and electronic applications, such as solar-cell devices, based on the photovoltaic effect^30^,^31^,^32^,^33^,^34^,^35^,^36^,^37^. Room temperature ferromagnetism has been found in undoped ZnO^38^,^39^,^40^,^41^,^42^,^43^, which is attributed to oxygen defects, especially singly ionized oxygen vacancies, V_o_^+^^39^,^41^,^43^. Here, we demonstrate that the ferromagnetism of ZnO thin film on Pt substrate could be enhanced by nonpolarized ultraviolet or violet light at room temperature. The enhancement of ferromagnetism in ZnO/Pt is possibly correlated with the enhancement in the amount of singly ionized oxygen vacancies, V_o_^+^, due to parts of doubly ionized oxygen vacancy, V_o_^++^, turning into singly ionized oxygen vacancies, accompanying the trapping of electrons from photogenerated electron-hole pairs. The built-in electric field due to the heterostructured ZnO/Pt is essential to the light-induced change in the ferromagnetism of ZnO. The built-in electric field not only separates the photo generated electron-hole pairs but also induces the displacement of electrons in ZnO. The enhancement of ferromagnetism could be observed when violet light (the photon energy with wavelength of λ = 380 nm is lower than the intrinsic band gap of ZnO, ∼3.37 eV) is used for illumination, as the effect of impurity energy states associated with the oxygen vacancies in ZnO effectively narrow the band gap^44^. We also find that the ferromagnetism for ZnO thin film on MgO substrate under light illumination does not significantly change, which is mainly due to the ultrafast recombination of photogenerated electron-hole pairs with the absence of a built-in electric field. Usually, a stronger photovoltaic effect in ZnO is obtained when oxygen annealing is performed^37^, in which the oxygen vacancies and carrier concentrations are reduced. However, in the present work, we find that the variation of ferromagnetism was not evident for ZnO thin film annealed in an oxygen atmosphere, even at the illumination of ultraviolet light with λ = 365 nm, which is mainly ascribed to the lack of sufficient doubly ionized oxygen vacancy (V_o_^++^) for the oxygen annealed ZnO. Our present work demonstrates a novel route to control the ferromagnetism of undoped semiconductors with nonpolarized light at room temperature.

## Results and Discussion

### Ferromagnetism of ZnO films on Pt substrate without light illumination

**Figure 1:**
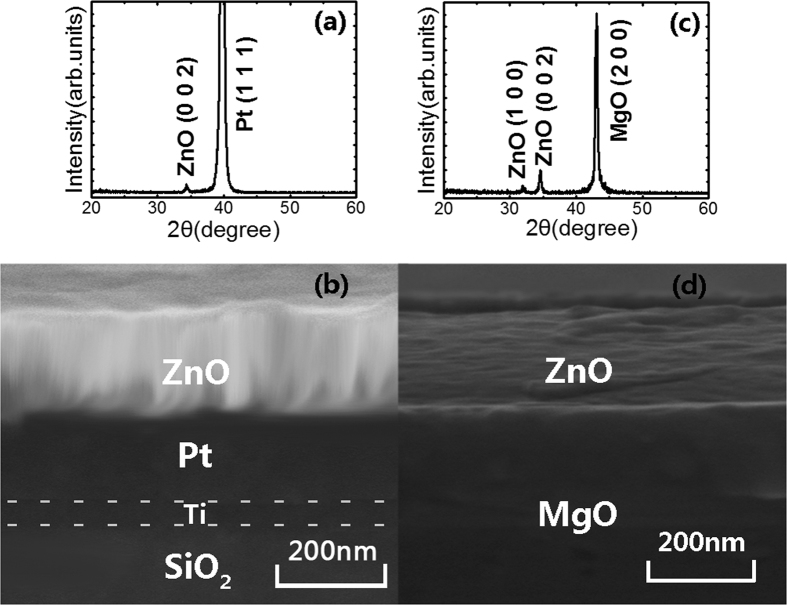
The (**a**) XRD pattern and (**b**) SEM cross-section photo of the ZnO film on the (1 1 1) Pt/Ti/SiO_2_/Si substrate. The (**c**) XRD pattern and (**d**) SEM cross-section photo of the ZnO film on the (1 0 0) MgO substrate.

**Figure 2:**
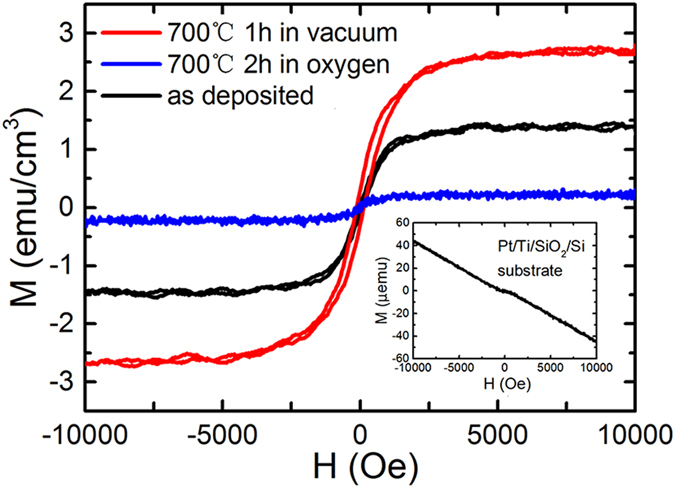
Room temperature ferromagnetism of ZnO films. The curves of the magnetic field (H) vs. the magnetization (M) after diamagnetism correction for the ZnO film on Pt/Ti/SiO_2_/Si substrate under three different conditions (as deposited, vacuum annealing and oxygen annealing) at room temperature, where the magnetic field is applied parallel to the surface of the film. The inset shows the M-H curve for the Pt/Ti/SiO_2_/Si substrate.

**Figure 3:**
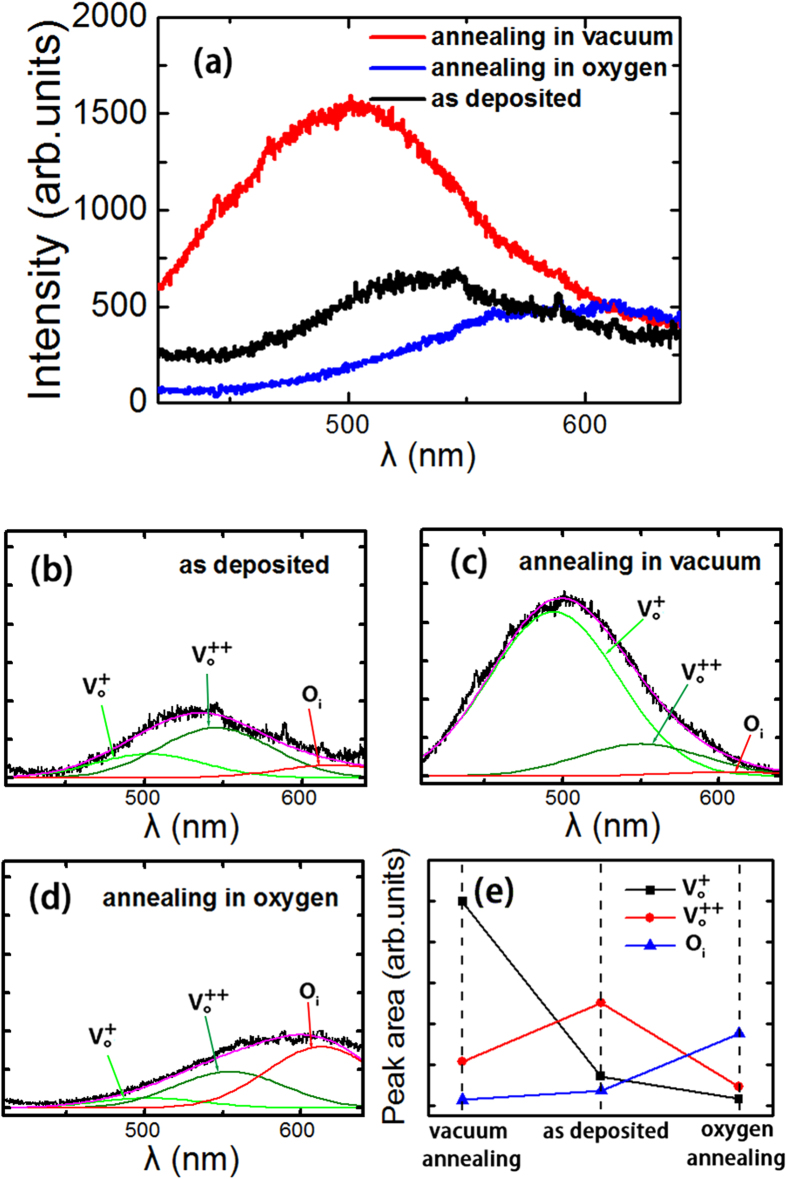
Photoluminescence of ZnO films. (**a**) The room temperature photoluminescence spectra of the ZnO films on Pt/Ti/SiO_2_/Si substrate under different conditions (as deposited, vacuum annealing and oxygen annealing). (**b**-**d**) Show the Gaussian fits of the photoluminescence spectra of the ZnO films with the three different conditions. (**e**) The peak areas of the photoluminescence emissions correlated with three oxygen-related defects (the singly ionized oxygen vacancies (V_o_^+^), the doubly ionized oxygen vacancy (V_o_^++^) and the intrinsic defects of oxygen interstitials (O_i_)) for the ZnO films under the three conditions (as deposited, vacuum annealing and oxygen annealing).

The X-ray diffraction (XRD) pattern of ZnO films grown on Pt/Ti/SiO_2_/Si by rf magnetron sputtering is shown in Fig. 1(a). A (0 0 2) peak from the hexagonal wurtzite ZnO structure can be observed in the figure. The morphology of the cross-section for the ZnO film grown on Pt/Ti/SiO_2_/Si substrate is shown in Fig. 1(b). The thickness of the ZnO film is approximately 200 nm. The magnetic field (H) dependence on the room temperature magnetization (M) after diamagnetism correction for the ZnO film as deposited on the substrate of Pt/Ti/SiO_2_/Si is shown in Fig. 2, where the magnetic field is applied parallel to the surface of the film (in-plane). The inset in the figure shows the M-H curve for the Pt/Ti/SiO_2_/Si substrate, which is diamagnetic (or nonmagnetic). The magnetic hysteresis loop of the ZnO film can be observed, showing the ferromagnetism at room temperature. The value of the saturation magnetization (Ms) is approximately 1.35 emu/cm^3^. The observed ferromagnetism of the undoped ZnO film belongs to d^0^ ferromagnetism induced by vacancies^38^,^39^,^40^,^41^,^42^,^43^. The role of cation or anion vacancies in introducing the d^0^ ferromagnetism is usually determined through the comparison of magnetization values between the air and vacuum or oxygen atmosphere annealing^24^,^25^,^27^. We have examined the effects of oxygen atmosphere annealing and vacuum annealing on the room temperature ferromagnetism of the ZnO film deposited on Pt/Ti/SiO_2_/Si. As shown in Fig. 2, the room temperature ferromagnetism of the ZnO film enhances after vacuum annealing at 700 °C for 1 hour, but reduces after oxygen annealing at 700 °C for 2 hours. In general, oxygen vacancies occur in high density for ZnO films when vacuum annealing, but are difficult to form in the case of annealing under a high pressure oxygen atmosphere. Our present results show that the observed room temperature ferromagnetism of the ZnO film deposited on Pt/Ti/SiO_2_/Si should originate from oxygen vacancies. Figure 3(a) shows the room temperature photoluminescence (PL) spectra for three types of ZnO films (as deposited, vacuum annealing and oxygen annealing) on Pt/Ti/SiO_2_/Si substrates. Gaussian fitting is performed on each spectrum for the three types of ZnO films, as shown in Fig. 3(b,c) and (d). Three peaks in the visible band frequently occur at approximately 504 nm, 550 nm, and 614 nm for the ZnO film. The emissions with 504 nm and 550 nm are related to the singly (V_o_^+^) and doubly ionized oxygen vacancies (V_o_^++^), and the emission approximately 614 nm originates from the intrinsic defects of the oxygen interstitials (O_i_)^39^. Figure 3(e) shows the peak areas of the photoluminescence emissions of the ZnO film with different conditions (as deposited, vacuum annealing and oxygen annealing). Generally, the oxygen vacancies of semiconductor oxide thin films tend to be filled by oxygen molecules from the environment after heat-treatment in an oxygen atmosphere. Comparing the cases of the as deposited and oxygen annealing samples shown in Fig. 3(e), it can be observed that both the oxygen vacancy contents, V_o_^++^ and V_o_^+^, in the ZnO film on the Pt substrate decrease after oxygen annealing. In contrast, the vacuum annealing leads to the sharp increase of V_o_^+^, as shown in Fig. 3(e). Comparing these results, one could determine that there is a positive correlation between the amount of V_o_^+^ vacancies and the strength of the saturation magnetization (Ms). It could be suggested that the ferromagnetism of the ZnO film originates from the singly ionized oxygen vacancies (V_o_^+^). Similar results have also been obtained for ZnO films deposited on quartz wafers or for ZnO nanoparticles^39^,^41^,^43^.

### Light control of ferromagnetism for ZnO film on Pt substrate

**Figure 4:**
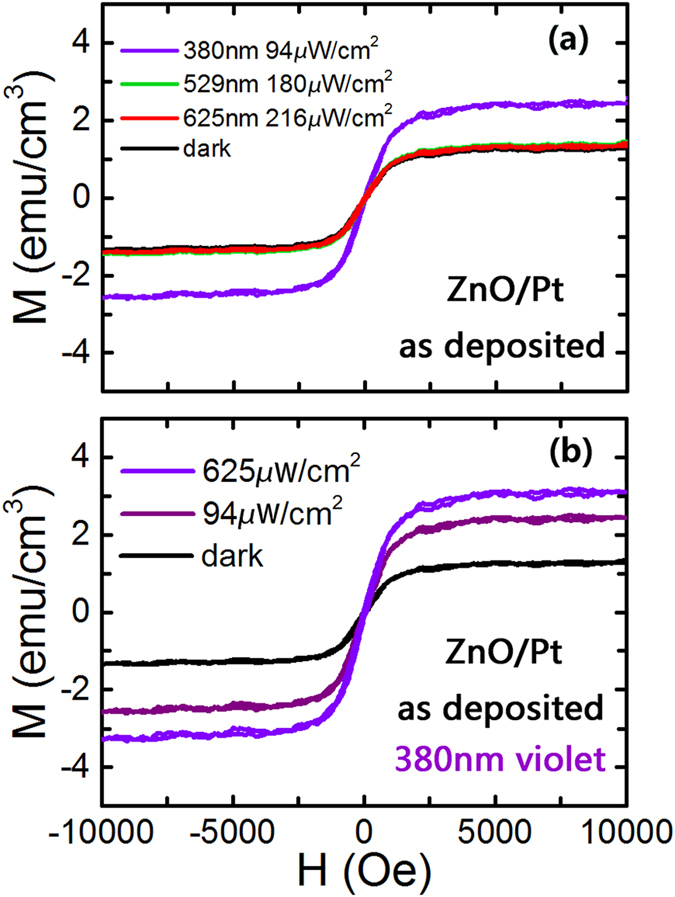
Light controlled ferromagnetism of ZnO film. (**a**) The in-plane ferromagnetism dependence on the light illumination with different wavelengths and intensities for the ZnO film on Pt/Ti/SiO_2_/Si substrate at room temperature. (**b**) The in-plane ferromagnetism dependence on the illumination intensity of violet light for the as deposited ZnO film on the Pt/Ti/SiO_2_/Si substrate at room temperature. The corrections of the diamagnetic signals from the substrate have been made.

In the following, we demonstrate that the ferromagnetism of ZnO films on Pt substrate can be controlled by light. Figure 4(a) shows typical M-H curves measured at room temperature for the ZnO film under different lighting conditions. Compared with the dark case, the saturation magnetization (Ms) has an evident increase under violet light illumination (λ = 380 nm, P_Light_ = 94μW/cm^2^). The Ms values of the ZnO film on Pt under green light illumination (λ = 529 nm, P_Light_ = 180μW/cm^2^) and red light (λ = 625 nm, P_Light_ = 216 μW/cm^2^) remain almost the same as compared with the dark case (without light illumination). We also find that the magnetization of the ZnO film on Pt depends on the illumination intensity. The saturation magnetization (Ms) of the ZnO/Pt increases with an increase in the illumination intensity of violet light, as shown in Fig. 4(b).

**Figure 5:**
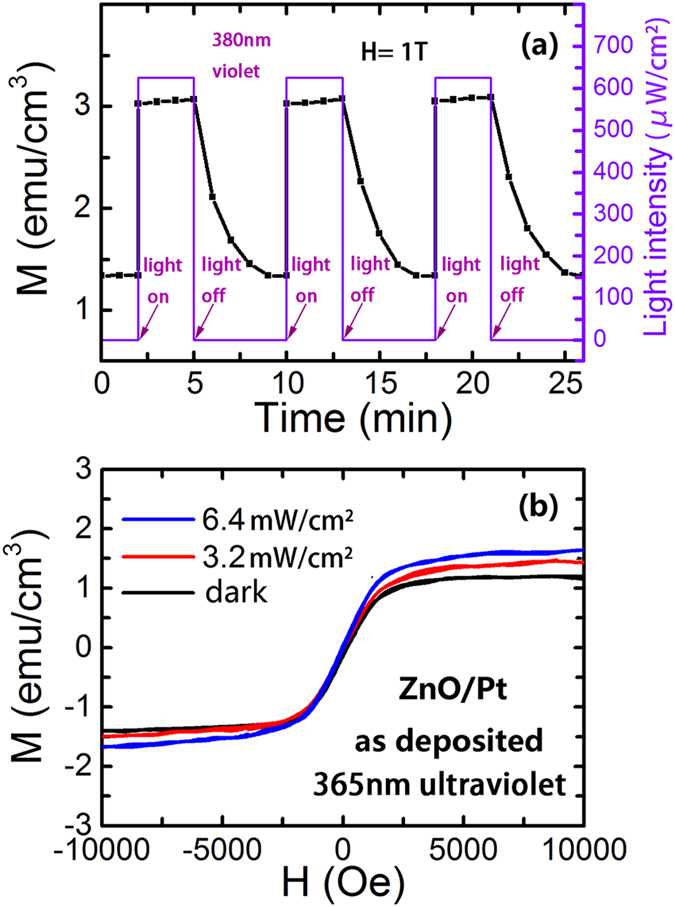
The dynamic response and recovery of magnetization. (**a**) The curves of the magnetization (M) vs. time (t) for the as deposited ZnO film on Pt/Ti/SiO_2_/Si substrate at room temperature under a square wave violet light (λ = 380 nm) with an illumination intensity of P_Light_ = 625 μW/cm^2^, where a magnetic field of 1 T is applied. (**b**) The curves of the magnetization (M) vs. the magnetic field (H) for the as deposited ZnO film on Pt/Ti/SiO_2_/Si substrate after being exposed outside under a higher illumination intensity of ultraviolet light (λ = 365 nm). The corrections of the diamagnetic signals from the substrate have been made.

The dynamic response and recovery of magnetization to violet light illumination (λ = 380 nm, P_Light_ = 625 μW/cm^2^) for the ZnO film on Pt substrate under an applied magnetic field H = 1 T is shown in Fig. 5(a).The ferromagnetism of the ZnO film on Pt substrate increases sharply from 1.35 emu/cm^3^ to 3.1 emu/cm^3^ when the violet light is on. When the light is turned off, the gradual reduction of the ferromagnetism lasts approximately 3 minutes, indicating that getting the device back to the initial magnetic state will take some time.

The after-effects of light illumination on the magnetic properties are also investigated. The magnetization loop of the ZnO film on Pt substrate is measured after the sample was first irradiated by a strong ultraviolet light (λ = 365 nm) for 3 minutes outside the magnetic measurement device. The starting time of the magnetic measurement is approximately 1 min after the light is turned off. As shown in Fig. 5(b), the saturation magnetization (Ms) of the ZnO/Pt can be adjusted by the presence of ultraviolet light (λ = 365 nm).

### The mechanism of light controlled ferromagnetism for ZnO films on Pt substrate

**Figure 6:**
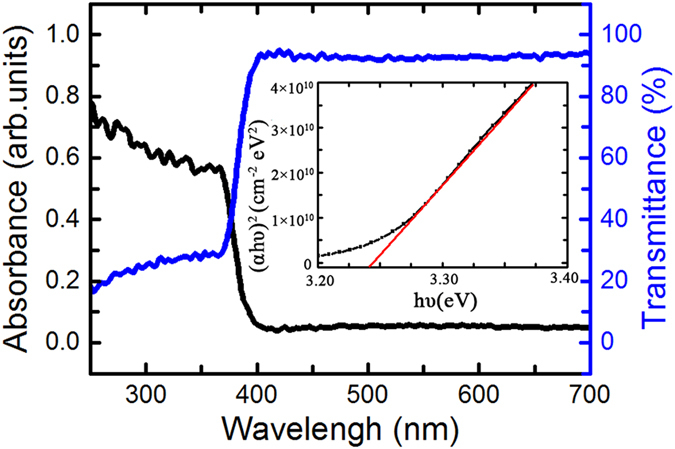
Optical properties of ZnO films. Transmittance and absorbance of the undoped ZnO film on sapphire substrate in the ultraviolet and visible-light regions. The inset shows a square of the absorption coefficient as a function of the photon energy.

Now, we discuss the mechanism of nonpolarized light controlled ferromagnetism for the ZnO film on Pt substrate. The photon energy larger than the band gap of 3.37 eV (i.e., λ < 368 nm) could induce the intrinsic absorption of light in ZnO. The electron-hole pairs can be generated in ZnO by the illumination of ultraviolet light (λ = 365 nm). However, various defects occur in ZnO film, and various (deep or shallow) impurity energy states are inevitably present in the gap, allowing the impurity absorption of light with lower frequencies, such as violet light (λ = 380 nm).The transmittance and absorbance of the ZnO film on sapphire substrate was measured, and the results are shown in Fig. 6. Based on the curve of (αhν) ^2^-hν (where α is the absorption coefficient, and hν is the photon energy), the band gap (E_g_) of our ZnO film can be derived^45^as 3.24 eV, narrower than the corresponding value of intrinsic ZnO. This is associated with effects of the oxygen vacancies in ZnO^44^. The photon energy with our violet light (wavelength λ = 380 nm) is larger than the band gap of our ZnO film; therefore, electron-hole pairs can be generated in the ZnO by the illumination of violet light. The derived penetration depths (d_0_ = 1/α) of different photons with λ = 365, 380, 529 and 625 nm are 166, 470, 2460 and 2730 nm, respectively. The thickness of the film is 200 nm, and the penetration depth of ultraviolet light (λ = 365 nm) is smaller than 200 nm. That means most of the ultraviolet light is absorbed. The penetration depth of violet light (λ = 380 nm) is larger than 200 nm, meaning limited violet light is absorbed. As for the green (λ = 529 nm) and red light (λ = 625 nm), the penetration depths are much larger than 200 nm, indicating that most of the light passes through the film.

It is well known that the interface of ZnO and Pt will form a Schottky contact because of their different work functions^46^. Since the work function of Pt is larger than ZnO, electrons in the contact surface of the ZnO film will flow to Pt and gather on the contact surface. Then, there will be a positive space charge region near the contact surface of the ZnO film due to the loss of the electrons. The direction of the built-in electric field inside the ZnO film is from the body to the surface. In the dark case, oxygen molecules in the air are adsorbed onto the surface of the ZnO film, forming the oxygen species O_2_^-^(ad) or O^-^(ad) by capturing electrons from the n-type semiconductor^47^,^48^,^49^, thereby creating a depletion layer near the outer-surface. When the ZnO film on Pt substrate is illuminated by an ultraviolet light, the photogenerated electron-hole pairs are separated by the built-in electric field. The holes will move to the depletion layer near the outer-surface induced by the built-in electric field. These holes are trapped by the surface adsorbed oxygen species O_2_^-^(ad) or O^-^(ad), and oxygen molecules are released. Meanwhile, electrons tend to move towards the Pt due to the built-in electric field. In this process of electron displacement, electrons could be captured by doubly ionized oxygen vacancy (V_o_^++^), which will make doubly ionized oxygen vacancies (V_o_^++^) become singly ionized oxygen vacancies (V_o_^+^). The increasing amount of singly ionized oxygen vacancies (V_o_^+^) enhances the ferromagnetism of the ZnO film. From Fig. 4(a), one can observe the enhancement of the ferromagnetism induced by the violet light (wavelength λ = 380 nm), where the photon energy of the light is lower than the intrinsic band gap of ZnO. These enhancements are associated with the presence of impurity energy states in the original band gap of ZnO and are associated with the oxygen vacancies, effectively narrowing the band gap^44^. As shown in Fig. 4(b), the ferromagnetism of ZnO film increases evidently under more powerful light illumination. It is understood that high illumination intensity light has more photons, which could excite more photogenerated electron-hole pairs and produce more singly ionized oxygen vacancies (V_o_^+^), enhancing the ferromagnetism.

When the light is turned off, the magnetization (M) of the ZnO/Pt drops to its dark value but with a longer recovery time of approximately 3 min, as shown in Fig. 5(a). In the present case, during the re-adsorption process of oxygen molecules on the ZnO outer-surface, the oxygen species O_2_^-^(ad) or O^-^(ad) would occur through trapping the electrons from the ZnO, accompanying the formation of holes in the ZnO. These holes would recombine with the electrons trapped by the V_o_^+^, accompanied by parts of V_o_^+^ turning into V_o_^++^. The longer recovery time is associated with the re-adsorption process of oxygen species on the outer-surface of the ZnO film. It has been found that the external environment of sample can affect the re-adsorption process of the oxygen species^48^. The oxygen atmosphere could accelerate the oxygen re-adsorption process. It can be expected that the recovery time could be shortened by increasing the oxygen pressure of sample environment.

**Figure 7:**
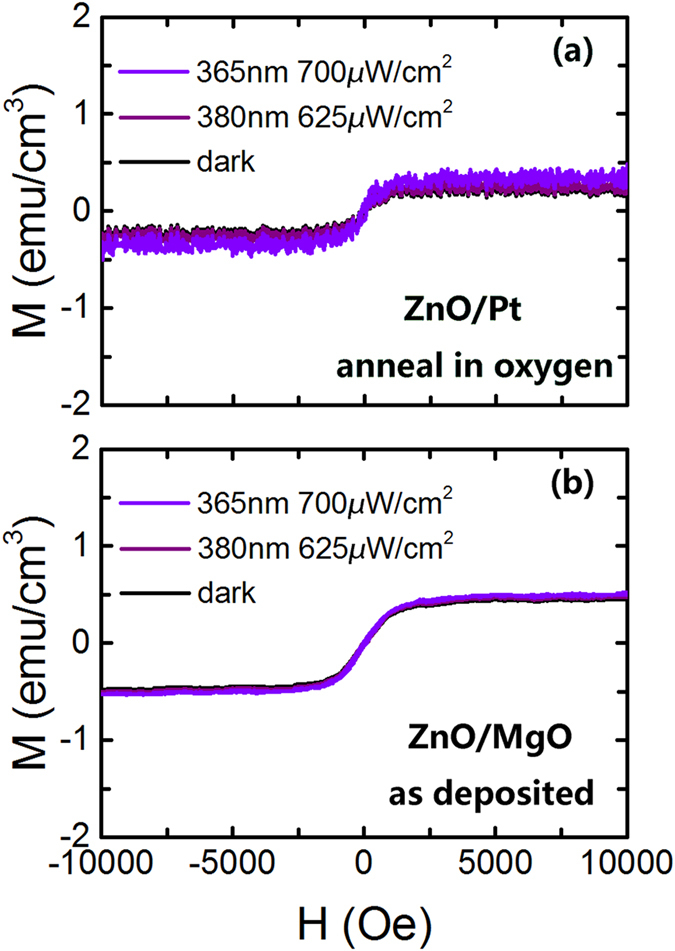
Invariable ferromagnetism of some ZnO films under light illumination. The in-plane magnetic hysteresis loops with and without the light illumination for (**a**) ZnO film on Pt/Ti/SiO_2_/Si substrates annealed in an oxygen atmosphere of 0.1 MPa at 700 °C for 2 hours and (**b**) the ZnO film on MgO substrate. The corrections of the diamagnetic signals from the substrate have been made.

As shown in Fig. 7(a), after the ZnO film on Pt substrate was annealed in an oxygen atmosphere of 0.1 MPa at 700 °C for 2 hours, the ferromagnetism remains nearly constant under violet light illumination and has only a small change under the ultraviolet light illumination. From Fig. 3(e), we can see a sharp decrease in the content of V_o_^++^ after annealing in the oxygen atmosphere. That means that a significant amount of oxygen vacancies, including V_o_^++^, were filled after annealing in the oxygen atmosphere, and there is not enough V_o_^++^ to trap the electrons from the photo generated electron-hole pairs. Simultaneously, the amount of V_o_^+^ does not vary widely when the oxygen annealed ZnO film is under the illumination of ultraviolet light. In addition, the oxygen annealing removes the impurity energy states and widens the band gap. The photon energy of violet light would thus be insufficient to excite the electron-hole pairs. This is why the ferromagnetism of the oxygen annealed ZnO film remains nearly constant under violet light illumination.

To explore more about light controlled ferromagnetism, ZnO films grown on (1 0 0) MgO single crystal substrates were investigated. The XRD patterns for the ZnO film on MgO substrate is shown in Fig. 1(c), and the morphology of the cross-section for the ZnO film on MgO is shown in Fig. 1(d). The ferromagnetism of the ZnO film on MgO is nearly constant under light illumination, as shown in Fig. 7(b). It appears that the built-in electric field due to the heterostructured ZnO/Pt is essential to the light induced changes in the ferromagnetism of ZnO. For the ZnO/MgO heterostructure, the built-in electric field is too small to reduce the recombination of electron-hole pairs. The fast recombination of the electron-hole pairs produces no obvious change of the saturation magnetization of the ZnO film on MgO substrate.

In summary, we have reported the light controlled ferromagnetism of ZnO films on Pt substrate at room temperature. The illumination of light with sufficiently high frequency photons could excite the photogenerated electron-hole pairs in the ZnO film. The enhancement of the ferromagnetism is possibly correlated with the enhancement in the quantity of singly ionized oxygen vacancies (V_o_^+^), due to the photogenerated electrons transferring to the doubly ionized oxygen vacancies (V_o_^++^). The built-in electric field due to the interface in the heterostructured ZnO/Pt not only separates the photogenerated electron-hole pairs but also induces the increased movement of electrons into the ZnO. When the light is turned off, the magnetization (M) of the ZnO/Pt drops to its dark value, but with a longer recovery time. This means that the ferromagnetism can be reversibly controlled by light with a sufficiently high frequency. The longer recovery time is associated with the re-adsorption process of oxygen species on the outer-surface of the ZnO film. To obtain an improved device, a large oxygen pressure of sample environment may be needed to accelerate the re-adsorption process of the oxygen species and reduce the recovery time. We expect that a similar phenomenon of light controlled ferromagnetism may also occur in other solar energy oxide thin films with noble metal substrates.

## Methods

ZnO films were grown on (1 1 1) Pt/Ti/SiO_2_/Si substrates (5 mm × 3 mm × 0.5 mm) using rf magnetron sputtering of pure ZnO in an argon-oxygen atmosphere at a 4:1 ratio. During the sputtering process, the working pressure was 1 Pa, and the substrate temperature was 600 °C. After deposition, the films were then annealed *in situ* at 600 °C for 60 min for better crystallization and then cooled to room temperature. The ZnO films grown on (1 0 0) MgO single crystal substrates (5 mm × 3 mm × 0.5 mm) and sapphire single crystal substrates (30 mm × 10 mm × 0.5 mm) were prepared using the same method and conditions. The crystal-structure of the films was examined with X-ray diffraction (XRD) at room temperature. The cross-sectional morphology of the ZnO films were observed by scanning electron microscopy (SEM). The photoluminescence (PL) spectra were also obtained at room temperature, where the excitation wavelength was 325 nm (He-Cd laser). Magnetization measurements were carried out using a VersaLab (Quantum Design). All measurements were carried out at room temperature. The light sources used in the experiment were light emitting diodes (LED) with different wavelengths (365 nm, 380 nm, 529 nm and 625 nm).For the direct measurement of the magnetic hysteresis loop for the ZnO film, the signals of the ZnO/substrate samples and the substrate were measured separately, and a subtraction was performed. For themeasurement of the magnetic hysteresis loop with an LED in the light-off state (dark conditions) in the VersaLab, the signal for the ZnO/substrate can be extracted from the measurement of the signals of the device with and without the ZnO/substrate. For the measurement of the magnetic hysteresis loop with a LED in the state of light-on (under the illumination condition) in VersaLab, the signal of the ZnO/substrate can be extracted from the measurement of the signals of the device with and without the ZnO/substrate. In this way, we could rule out both the effects of the LED and the current of the LED in our final results. The after-effects of light illumination on the magnetic properties were also determined, where the magnetic hysteresis loops were directly measured (without LED) with the VersaLab, after the illumination of the LED with the high illumination intensity on the sample outside of the VersaLab. In our measurements, the signal of the ZnO film was extracted after correcting for the substrate signal.

## Additional Information

**How to cite this article:** Xie, J. *et al*. Light Control of Ferromagnetism in ZnO Films on Pt Substrate at Room Temperature. *Sci. Rep.* **7**, 45642; doi: 10.1038/srep45642 (2017).

**Publisher's note:** Springer Nature remains neutral with regard to jurisdictional claims in published maps and institutional affiliations.
